# Structural, Morphological and Thermal Properties of Cellulose Nanofibers from Napier fiber (*Pennisetum purpureum*)

**DOI:** 10.3390/ma13184125

**Published:** 2020-09-17

**Authors:** Revati Radakisnin, Mohd Shukry Abdul Majid, Mohd Ridzuan Mohd Jamir, Mohammad Jawaid, Mohamed Thariq Hameed Sultan, Mohd Faizal Mat Tahir

**Affiliations:** 1 Faculty of Mechanical Engineering Technology, Universiti Malaysia Perlis (UniMAP), Pauh Putra Campus, Arau, Perlis 02600, Malaysia; revark_1990@yahoo.com (R.R.); ridzuanjamir@unimap.edu.my (M.R.M.J.); 2 Faculty of Electronic Engineering Technology, Universiti Malaysia Perlis (UniMAP), Pauh Putra Campus, Arau, Perlis 02600, Malaysia; 3 Laboratory of Biocomposite Technology, Institute of Tropical Forestry and Forest Products (INTROP), Universiti Putra Malaysia, Serdang, Selangor 43400, Malaysia; jawaid@upm.edu.my; 4 Department of Aerospace Engineering, Faculty of Engineering, Universiti Putra Malaysia, Serdang, Selangor Darul Ehsan 43400, Malaysia; 5 Aerospace Malaysia Innovation Centre (944751-A), Prime Minister’s Department, MIGHT Partnership Hub, Jalan Impact, Cyberjaya, Selangor Darul Ehsan 63000, Malaysia; 6 Center for Integrated Design of Advanced Mechanical Systems (PRISMA), Faculty of Engineering and Built Environment, Universiti Kebangsaan Malaysia, Bandar Baru Bangi, Selangor 43600, Malaysia; mfaizalmt@ukm.edu.my

**Keywords:** Napier fiber, cellulose nanofiber, crystallinity, morphology properties, thermal properties

## Abstract

The purpose of the study is to investigate the utilisation of Napier fiber (*Pennisetum purpureum*) as a source for the fabrication of cellulose nanofibers (CNF). In this study, cellulose nanofibers (CNF) from Napier fiber were isolated via ball-milling assisted by acid hydrolysis. Acid hydrolysis with different molarities (1.0, 3.8 and 5.6 M) was performed efficiently facilitate cellulose fiber size reduction. The resulting CNFs were characterised through Fourier-transform infrared spectroscopy (FTIR), X-ray diffraction (XRD), thermogravimetric analysis (TGA), particle size analyser (PSA), field-emission scanning electron microscopy (FESEM), atomic force microscopy (AFM), and transmission electron microscopy (TEM). The FTIR results demonstrated that there were no obvious changes observed between the spectra of the CNFs with different molarities of acid hydrolysis. With 5.6 M acid hydrolysis, the XRD analysis displayed the highest degree of CNF crystallinity at 70.67%. In a thermal analysis by TGA and DTG, cellulose nanofiber with 5.6 M acid hydrolysis tended to produce cellulose nanofibers with higher thermal stability. As evidenced by the structural morphologies, a fibrous network nanostructure was obtained under TEM and AFM analysis, while a compact structure was observed under FESEM analysis. In conclusion, the isolated CNFs from Napier-derived cellulose are expected to yield potential to be used as a suitable source for nanocomposite production in various applications, including pharmaceutical, food packaging and biomedical fields.

## 1. Introduction

The fibers of Napier grass, also commonly known as *Pennisetum purpureum* (PP), are made up of 46% cellulose, 34% hemicellulose, and 20% lignin [1]. In addition, it requires only a minimal supply of nutrients for growth. In order to eliminate impurities and non-cellulosic material, an alkaline treatment was implemented in most studies, using sodium hydroxide (NaOH) (4–20%) with 1–5 h of continuous stirring. The treated fibers were then washed with distilled water until the pH was neutralised and then dried in an oven overnight at 50 °C [2]. Eliana et al. reported that Napier fibers with alkaline pre-treatment yielded the highest percentages of lowering sugars and ethanol [3]. It was reported that the delignification of Napier grass was carried out by alkaline treatment with different concentration from 0.5 to 10.5 wt.%, thus resulting in 80.59% cellulose and removal of 93.78% lignin [4]. Ridzuan et al. recommended Napier fiber as a potential reinforcement material in polymer composites [5]. Devin and Samir also recommended cellulosic fibers from Napier grass can be used as supporting material for biofuel productions owing to the high moisture content of the cellulose [6]. According to the previous studies, alkaline pre-treatment is a preferred method for hemicellulose and lignin removal; thus, cellulose retrieval from this method is promising. Alkaline post-treatment is also proposed in this study to eliminate any remaining hemicellulose and lignin further.

Recently, the development of nanocellulose from cellulose source as a preferred reinforcement for nanocomposites has generated significant research interest on the utilisation of natural fibers owing to their outstanding mechanical properties, sustainability, affordability, low environmental impact, and relatively good specific features. There are several literature reviews on the expansion of nanocellulose from cellulose source, via various methods. V. K. Baheti et al. had conducted dry and wet ball milling of CNF from jute fiber wastes, which resulted in nanoparticles sized below 500 nm with a small amount of contamination imported from milling media [7]. Morais et al. studied on CNF from different types of cotton fibers using acid hydrolysis with 6.5M of sulphuric acid at 45 °C. They concluded that extraction yield, sulfonation efficiency, and thermal stability varies according to the type of cotton fibers [8]. Zhijun et al. obtained CNF from bamboo fiber from a combination of mechanical treatment, enzyme activation, carboxymethylation, and ultrasonic homogenisation [9]. They found that extraction yield, surface charge and carboxymethylation reaction was enhanced through the existing preparation procedures. Based on these reviews and the related studies regarding the nanofibers from cellulose, it can be concluded that preparation and isolation plays a crucial part in producing a good cellulose nanofiber with enhanced structural and mechanical properties.

Nanocellulose is described as a natural nanomaterial that is either a product of or is extracted from native cellulose sources, such as plants [10]. The obtained cellulose from plants can be further reduced into smaller cellulose fragments that are either micro-sized or nano-sized. Nanocellulose, with its nano-sized diameter, has many advantages such as a high surface area, good strength and stiffness, excellent chemical reactivity, and being low in density [11]. Recently, they have been widely used as cellulose nanofibers (CNF), or cellulose nanocrystals (CNC), attracted positive attention in many industries such as the automotive [12], biomedical [13,14,15], and pharmaceutical industries [10], and have also found use as reinforcements in polymeric nanocomposites [16,17]. From previous studies, nanocellulose fibers have been successfully extracted from cellulose sourced from softwood [18], cotton [8,19,20], roselle [21], jute fiber [22], banana peel [23,24], and bamboo [25]. Nevertheless, it is crucial to acknowledge that cellulose characteristics, such as the structural morphology, degree of crystallinity, degree of polymerisation, and size, depend upon the source from which the cellulose was extracted and not just on the extraction method employed [26,27].

CNF, also recognised as cellulose nanofibril or nanofibrillated cellulose, is the entangled, long, and flexible nanocellulose that can be produced from cellulose fibers that undergo mechanical processes in which both shear and impact forces are used [28]. The key feature is the size of the cellulose nanofiber, which is typically less than 100 nm in diameter and several micrometres in length [2]. The most commonly used mechanical techniques used to produce CNFs include microfluidisation, ultrasonication, high-pressure homogenisation, ball milling, and micro-grinding [18,29,30,31]. However, high-energy consumption is a major drawback of the mechanical processes used for the production of CNFs, where the production of fine particles sizes down to the range of nanometers was transformed by this high-energy ball milling by increasing rotational speed up to 1100 rpm for a couple of hours [32,33,34]. To overcome this shortcoming, the mechanical processes are combined with chemical pre-treatment to reduce the energy consumption, since pre-treated cellulose fibers are easily fibrillated and also prevent clogging [35,36].

Recently, various researches have illustrated the synthesis and isolation process of cellulose nanofibers by combining both chemical and mechanical treatments effectively. Leticia et al. had researched isolation of CNFs from cassava root baggase and peelings using 30, 40, and 50% of sulfuric acid, and the hydrolyzed suspensions were further homogenized to reduce the size and to disperse the CNFs using ultrasound. They conclude that the isolation of CNFs is inexpensive and nanofibers with excellent properties can be obtained [37]. The extraction of CNFs from pineapple leaf fibers (PALF) had been studied by Lakshmipriya et al. where the fibers were subjected to acid hydrolysis with 50% of sulfuric acid, and further ball-milled the suspension for 1.5 and 3 h. They found that the extraction process is environmentally sustainable and economical for the fabrication of good-quality CNF [38]. Lastly, Kittiya et al. had investigated the effect of sulfuric acid concentration on sugarcane bagasse fibers. The cellulose was ball-milled for 12 h with acid hydrolysis where the concentrations of sulfuric acid are 0, 20, and 40%. The researcher illustrated that the extracted CNFs has greater absorption than raw cellulose, whereas the crystallinity of the isolated CNFs was higher than untreated cellulose [39]. 

From the literature review conducted, it is evident that there are fewer reported studies on the optimisation of cellulose extraction from Napier fiber as a resource for developing CNFs. The objective of this research is to isolate and prepare cellulose nanofibers from Napier fiber by combining mechanical processing with acid hydrolysis and ultrasonication. The structural morphology, thermal stability and crystallinity of the isolated CNFs were then characterised. Hence, this study is to provide crucial information in preparing a future study on CNF from Napier fiber to look its appropriateness in synthesising nanocomposites for application advancements.

## 2. Materials and Experimental Methods

### 2.1. Materials

Raw Napier grass was obtained from a nearby plantation at Bukit Kayu Hitam, Kedah; northern peninsular Malaysia. Sulfuric acid (H_2_SO_4_) (purity ≥ 98 wt%, *Mm* = 98 g/mol), and sodium hydroxide (NaOH) were obtained from Fisher Scientific. All other chemicals used in this work were of reagent grade and purchased from local suppliers. De-ionised water was used throughout the experiments.

### 2.2. Extraction and Preparation of Napier Fibers

Water retting was employed in this study to remove the Napier fibers from the stem internodes [5]. The stems were at first cleaned and crushed into small pieces using a mallet before being soaked in a tank filled with water for 4 to 6 weeks to ease the separation process. The Napier fibers were manually extracted from the bast and cleaned with distilled water. The extracted fibers were sun-dried to remove the excess moisture content, which may exist within the fibers. Subsequently, the dried fibers were ground and sieved to convert the Napier fibers into fine particles (<63 µm).

### 2.3. Preparation of Cellulose Nanofibers from Napier Fibers (CNF-PP)

The ground fibers were further treated with 12% NaOH at 25 °C with an immersion time of 120 min. A liquor ratio of 20:1 was used in this experiment to eliminate the hemicellulose, lignin, and impurities from the fibers. Subsequently, the treated fibers were cleaned with distilled water and air-dried at 25 °C. The obtained fibers that maximally reduced hemicellulose and lignin contents were acid hydrolysed by treating 10 g cellulose with 400 mL of 1.0 M, 3.8 M and 5.6 M H_2_SO_4_, respectively, at 80 °C for 60 min under continuous mechanical stirring. The resulting mixture after each acid hydrolysis was repeatedly centrifuged at 3000 rpm for 25 min. The suspension was further neutralised for three days using a dialysis membrane (30 mm diameter, MWCO 12,000–14,000, SERVAPOR, SERVA Electrophoresis GmbH, Heidelberg, Germany) with distilled water to reach a neutral pH. Post-treatment of Napier fibers was then conducted to eliminate the remaining impurities from the fibers. The fibers were treated with a 4% NaOH solution at 80 °C for a further 60 min with continuous stirring. The suspension was centrifuged and washed using distilled water until the pH of the suspension became neutral.

After the chemical treatment, milling of the Napier fibers was carried out by a planetary ball milling machine (Bench-Top Planetary Automatic Ball Mill, MSK-SFM-1, MTI, Richmond, VA, Canada) in a zirconia bowl using zirconia balls with a diameter of 15 mm for 180 min of wet milling in de-ionised water. The ball to material ratio (BMR) of the container loaded with fibers was 10:1, and the speed of rotation of the container was set at 840 rpm. The resulting suspension was further sonicated for 15 min in an ultrasonicator (Branson Digital Signifier, Emerson, Danbury, CT, USA) at an amplitude of 40% with 8 s of pulse on, and 4 s of pulse off. Ultrasonication was done in an ice-bath to prevent heat-up where desulfation can occur due to the presence of sulfate groups on the fibers. Finally, the suspension was freeze-dried and stored for further characterisation and use as reinforcement fillers in biocomposites. A summary of the selected processing parameters for the synthesis of CNFs from Napier fibers is given in Table 1.

### 2.4. Characterisation

#### 2.4.1. Fourier Transform Infrared Spectroscopy (FTIR)

Infrared spectra were used to identify the chemical structure of the lignocellulosic elements present in the samples. Fiber spectra were characterised using a Perkin Elmer Spectrum Version 10.5.2 spectrophotometer with a total of 42 scans in the range of 4000–600 cm^−1^. A resolution of 4 cm^−1^ was used in this work.

#### 2.4.2. X-Ray Diffraction (XRD)

XRD was used to analyse the phase composition of the samples using a D2 Phase Bruker diffractometer with Cu-Kα radiation at 30 mA and 40 kV. Scattered radiation was recorded in the interval 10° ≤ 2θ ≤ 80° at a scan speed of 4°/min with a step time of 0.24 s and a step size of 0.02° 2θ. The crystallinity index (CrI) of the cellulose, CrI, was calculated using the empirical method [40], as illustrated in Equation (1)

(1) $$\mathrm{CrI}\left(\%\right)=\frac{I_{200}-I_{\mathrm{am}}}{I_{200}}\times 100$$

where I_200_ represents the crystalline peak corresponding to the intensity at approximately 22.4°, and I_am_ is the amorphous peak corresponding to the intensity at approximately 18.32°.

#### 2.4.3. Particle Size Measurements

The average hydrodynamic particle size of the fabricated CNFs in aqueous suspension was determined using a particle size analyser (PSA) (Malvern Instruments, Nano Z.S., Malvern Panalytical Ltd., Malvern, UK). The CNF particles were analysed in the range from 0.6 to 6000 nm under the following conditions: a temperature of 25 °C, viscosity of 1,2000 mPas, and scattering angle fixed at 90°. The sonicated CNF suspensions were all evaluated in triplicate. 

#### 2.4.4. Thermogravimetric Analysis (TGA)

Thermal stability was measured using a thermogravimetric analyser (Shimadzu DTG 60H, Kyoto, Japan). Samples weighing 10 mg were placed in an alumina crucible and evaluated by increasing the temperature constantly from 30 to 950 °C. All measurements were made under the flow of the dynamic nitrogen gas carrier with a flow rate of 20 mL/min. The loss of weight was obtained from the TGA curve of a plot of weight loss (%) versus temperature (°C).

#### 2.4.5. Field Emission Scanning Electron Microscopy (FESEM)

The surface morphology of the CNFs was investigated using FESEM, performed on a ZEISS MERLIN, Jena, Germany field emission scanning electron microscope at an acceleration voltage of 20 kV. The freeze-dried CNF samples were cut into a thin layer and mounted individually onto a sample holder. In order to avoid unwanted charging, the surface of each of the prepared samples was further sputtered with a thin layer of gold before FESEM was initiated. All measurements of the CNFs from FESEM images were obtained using the Smart Tiff software.

#### 2.4.6. Atomic Force Microscope (AFM)

The structural morphology and topography of the CNFs from Napier fibers were analysed by AFM. The samples were examined in a Park Systems NX-10 microscope, Suwon, Korea. A droplet of the CNF suspension was deposited on a glass slide, allowed to dry at 25 °C overnight, and then analysed by AFM. The AFM experiments were conducted in the tapping mode, at 25 °C. The scan was carried out at a rate of 1 Hz, with an image resolution of 0.015 nm, attached to a silicon cantilever spring constant of between 25 and 50 N m^−1^, followed by a resonance frequency up to 10.5 kHz. The width of the CNFs was analysed from the AFM images using XEI software.

#### 2.4.7. Transmission Electron Microscopy (TEM)

The TEM measurements were performed with an FEI Talos L120C microscope, Thermo Fisher Scientific, Oregon, OR, USA. CNF from Napier fibers was further diluted using ethanol before a drop of the diluted Napier nanofiber suspension was deposited onto a thin carbon-coated copper grid. The prepared samples were allowed to dry at 25 °C and observed and analysed through TEM at an acceleration voltage of 80 kV. The average diameter of the CNFs was calculated from the obtained TEM images using Image J software. 

## 3. Results and Discussions

### 3.1. Morphology of CNFs from Napier

**Figure 1:**
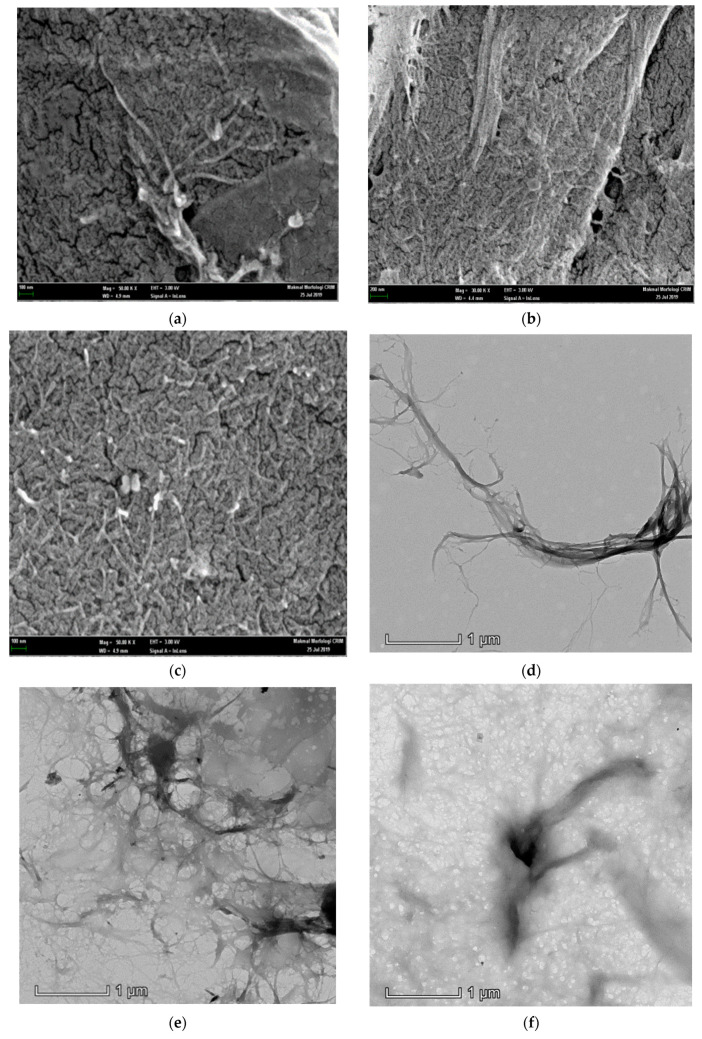

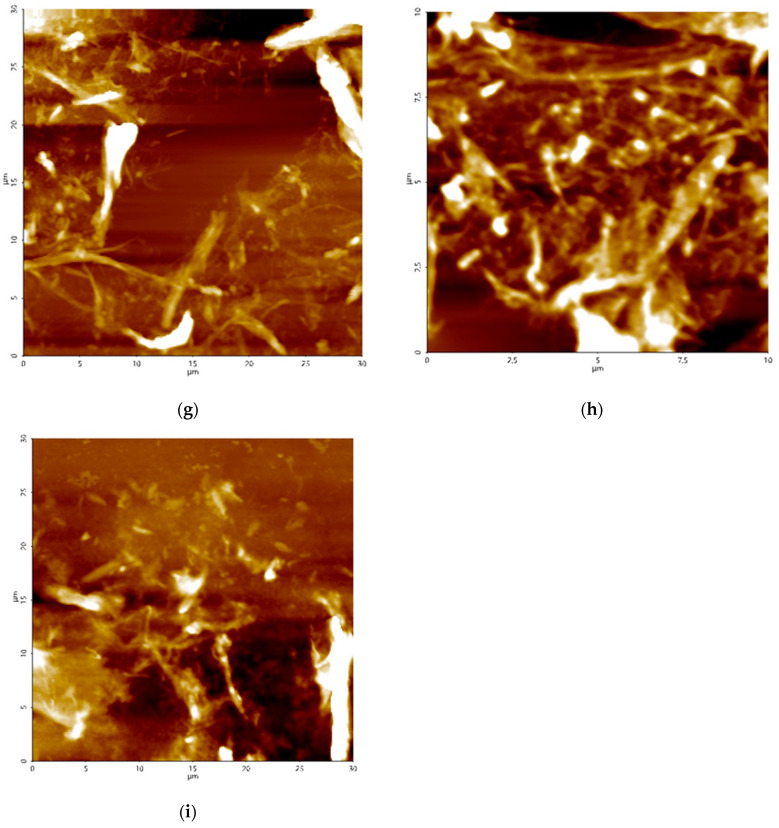
Field-emission scanning electron microscopy (FESEM) (**a**–**c**), transmission electron microscopy (TEM) (**d**–**f**), and atomic force microscopy (AFM) (**g**–**i**) results for CNF-PP_1.0M_ (**a**,**d**,**g**), CNF-PP_3.8M_ (**b**,**e**,**h**) and CNF-PP_5.6M_ (**c**,**f**,**i**).

Morphological studies on CNF-PP_1.0M_, CNF-PP_3.8M_ and CNF-PP_5.6M_ were carried out using FESEM, TEM, and AFM, and the obtained results are shown in Figure 1. Table 2 displays the morphological characterisations, where the diameters of CNF-PP_1.0M_, CNF-PP_3.8M_ and CNF-PP_5.6M_ are recorded. Figure 1a–c shows the FESEM micrographs of CNF-PP_1.0M_, CNF-PP_3.8M_ and CNF-PP_5.6M_, respectively, displaying their homogeneity and nanometric dimensions. The diameters of all three samples were computed by analysing the image using the processing software, Image J. The smallest and biggest diameters of CNF-PP_1.0M_, CNF-PP_3.8M_ and CNF-PP_5.6M_ were 49.93 and 167.6 nm, 25.46 and 34.67 nm, then 16.10 and 34.95 nm, respectively. Figure 1a–c shows the structure of the nanofibers after the acid hydrolysis and ball milling treatments. It can be seen that both treatments assist the separation of the bundle of fibers into individual fibers and fibrous network, thus leading to a significant reduction in their diameter size. The nanofibers of CNF-PP_1.0M_, CNF-PP_3.8M_ and CNF-PP_5.6M_ were visible in the freeze-dried samples. Nano-fragments may tend to agglomerate with nanofibers by an interfacial hydrogen bonding. 

Figure 1d–f shows TEM images of CNF-PP_1.0M_, CNF-PP_3.8M_ and CNF-PP_5.6M_. Based on the TEM analysis, CNF-PP_1.0M_ displayed an individuated, long, and fine nanofiber structure instead of a fibrous network structure; whereas for CNF-PP_3.8M_ and CNF-PP_5.6M_, a network-like fiber structure was observed. The ability to form a network of fibers is an essential criterion for nanofibers to develop into an effective reinforcement material when applied to biocomposites. Moreover, the CNF-PP_1.0M_ contained diameter sizes ranging from 5.04 to 90.67 nm compared to those of CNF-PP_3.8M_ and CNF-PP_5.6M_, which had the range 4.40–22.62 and 5.67–13.70 nm, respectively (Table 2). It is also important to note that the lengths of the obtained CNFs were estimated to be in the micrometric scale. The reduction in the diameter of CNF-PP_1.0M_, CNF-PP_3.8M_ and CNF-PP_5.6M_ were caused by the removal of lignin, hemicellulose, and the non-cellulosic parts of *Napier*, through the alkaline treatments (pre- and post-), acid hydrolysis and ball milling treatments. The morphology results of the CNF-PP_1.0M_, CNF-PP_3.8M_ and CNF-PP_5.6M_ samples in this study correlated well with the dimensions of the CNFs from kenaf (1–40 nm) [41], banana peel (20–50 nm) [24], cotton (20–80 nm) [19], and pineapple leaf fibers (20–50 nm) [38].

The CNFs obtained after acid hydrolysis and ball milling were analysed by AFM to determine their structure and diameter. The AFM image in Figure 1g displays the structural morphology of CNF-PP_1.0M_. It can be observed that the cellulose is nano-sized with a diameter ranging from 26.44 to 192.50 nm. Figure 1h shows the structural morphology of CNF-PP_3.8M_, with a diameter ranging from 19.64 to 53.28 nm, whereas Figure 1i displayed a diameter ranging from 10.50 to 38.74 nm. CNF-PP_3.8M_ and CNF-PP_5.6M_ presented more densely packed fibrous networks of cellulose nanofiber than CNF-PP_1.0M_ (Figure 1b,c), but both CNF-PP_1.0M_ and CNF-PP_3.8M_ displayed network-like structures of CNFs which concur with the TEM results. The diameter for CNF-PP_1.0M_, CNF-PP_3.8M_ and CNF-PP_5.6M_ obtained from AFM were larger than the diameter ranges from FESEM and TEM analysis. This is similar to the AFM results obtained for the isolation of CNFs from softwood pulp using TEMPO techniques [18]. A similar outcome was observed by Niu et al., where the extraction of CNC from microcrystalline cellulose using acid hydrolysis assisted by ultrasonication degraded the impurities and decreased the diameter of the CNC, breaking them down into nano-sized particles [42].

By comparing the structural morphology results obtained from FESEM, TEM, and AFM, it can be concluded that of all the microscopic analyses, TEM presented the clearest insight into CNF morphology, with a diameter size between 5.04 and 90.67 nm for CNF-PP_1.0M_, 4.40 and 22.62 nm for CNF-PP_3.8M,_ and 5.67 and 13.70 nm for CNF-PP_5.6M_. Additionally, all three samples displayed a fibrous network structure; hence, the structural morphology showed an increase in specific surface area compared to extracted Napier fibers. On the other hand, it was noticeable that the diameter measured using AFM was slightly larger than that from the FESEM and TEM analyses. This could be due to the broadening effect in AFM, where the broadening of the sample width depends on its physical properties and position concerning the tip radius. Another crucial aspect that might affect AFM is the placement of the nanofibers on the holder that may lead to random measurements due to the irregular shape of the nanofibers [43]. Thus, the combination of acid hydrolysis and ball milling further proved that when the impurities covering the CNFs were effectively removed, a reduction from micro-sized raw cellulose, composed of bundles of fibers, into nano-sized CNFs occurs.

### 3.2. Particle Size Measurement

**Figure 2:**
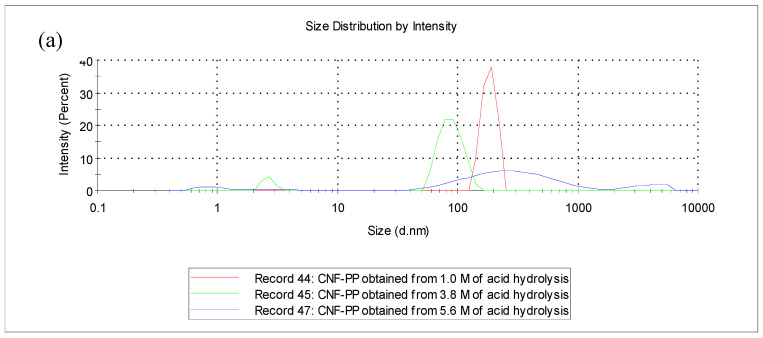

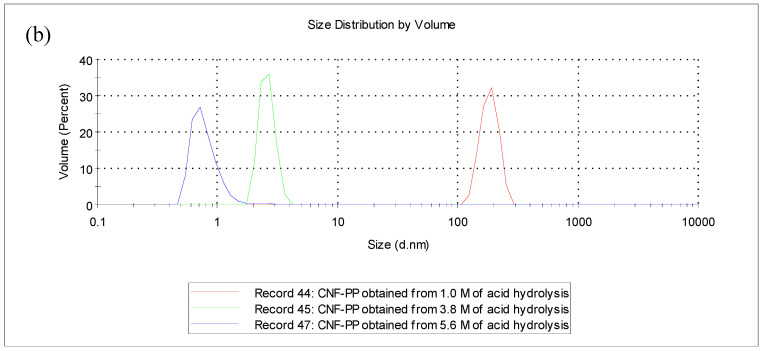
Particle size distribution by particle size analysis (PSA) (**a**) intensity-weighted distribution and (**b**) volume-weighted distribution of CNF-PP_1.0M_, CNF-PP_3.8M_ and CNF-PP_5.6M_.

Particle size analysis (PSA) is a method used to obtain the particle size of CNFs in suspension that undergoes Brownian motion generated by thermally induced collisions between the CNF particles and solvent particles. The measured particle size, often called a hydrodynamic diameter, which indicates the way that the CNF particle diffuses within a fluid. It is essential to understand that PSA is based on the Stokes–Einstein equation, where the measurements refer to spherical particles and the orientation of the CNFs in suspension profoundly influences the particle size values obtained [44]. The size distribution of CNF-PP_1.0M_, CNF-PP_3.8M_ and CNF-PP_5.6M,_ as measured by particle size analysis, is shown in Figure 2a,b.

Figure 2a shows the intensity weighted distribution of the CNF-PP_1.0M_, CNF-PP_3.8M_ and CNF-PP_5.6M_ samples as detected by laser diffraction. CNF-PP_1.0M_ showed a single peak with a value of approximately 525 nm. CNF-PP_3.8M_ displayed two peaks, minor peak with a mean value of 3 nm and maximum intensity of approximately 8% and a major peak with a mean value of 89 nm and maximum intensity of approximately 92% whereas for CNF-PP_5.6M_, three different peaks were observed. The first with an average size of 1 nm and an intensity of approximately 6%, the second with a mean value of 344 nm and maximum intensity of approximately 81%, and the third with a mean value of 3800 nm and maximum intensity of approximately 11%. Based on the results, CNF-PP_5.6M_ possesses larger particle sizes, indicating that particles aggregated in suspension more rapidly than CNF-PP_1.0M_ and CNF-PP_3.8M_ particles. This is the case for particles that possess high hydrophobic characteristics, i.e., samples containing less hydroxyl groups tend to aggregate easily [45].

Concerning the volume-weighted distribution (Figure 2b), the majority of the particles were in an equivalent volume range from 0.5 to 5 nm for CNF-PP_5.6M_, from 1 to 10 nm for CNF-PP_3.8M_ and from 100 to 500 nm for CNF-PP_1.0M_. CNF-PP_1.0M_ proved that low acid hydrolysis molarities were not sufficient to obtain nano-sized cellulose particles, even though the variables for the mechanical treatments were improved throughout the experiment and finally led to the breakdown of the weaker interactions between the crystalline region and amorphous region of CNFs [46]. When comparing particle size distribution data, these results show that the intensity-weighted distribution and volume-weighted distribution produce considerably different particle size measurements. However, it is crucial to understand that the intensity-weighted distribution was obtained using the intensity of the light scattered by the particle fractions, whereas the volume-weighted distribution was measured using image analysis. Analysing the particle size distribution by laser diffraction yields the most accurate results [37].

### 3.3. XRD Analysis

**Figure 3:**
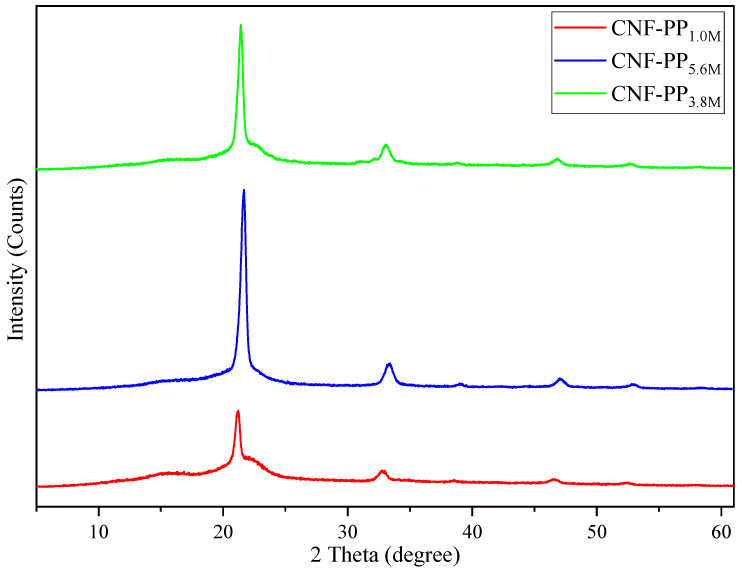
X-ray diffraction patterns of CNF-PP_1.0M_, CNF-PP_3.8M_ and CNF-PP_5.6M_.

X-ray diffraction patterns of the cellulose nanofibers were analysed to determine the influence of the molarity of acid hydrolysis on the crystallinity, where Segal’s method was used to calculate the crystallinity index, CrI. Figure 3 displays the corresponding X-ray diffractograms obtained for CNF-PP_1.0M_, CNF-PP_3.8M_ and CNF-PP_5.6M_. The diffractograms for the CNFs present intense peaks at around 16.2°, 22.6°, and 34.3°, which reflect on the crystallographic planes of (110), (200), and (004), respectively. Based on the literature, these crystallographic planes confirm that the CNF samples are in the crystal structure known as cellulose Iβ [25]. These results also confirm that the crystal integrity of the CNFs has been maintained through the ball milling and chemical treatment procedures [21].

As seen in Figure 3, the crystallinity of CNFs from Napier fibers was determined at the end of the isolation process. After ball milling, significant differences on the diffraction peaks of the CNFs were observed for CNF-PP_1.0M_, CNF-PP_3.8M_ and CNF-PP_5.6M_. As illustrated in Table 3, the crystallinity values of CNF-PP_1.0M_, CNF-PP_3.8M_ and CNF-PP_5.6M_ were 58.90, 65.18, and 70.67%, respectively. These results evidently indicate an increase in the crystallinity index (CrI) owing to the increase in the acid hydrolysis molarities, as stated by several authors [25,47]. CNF-PP_5.6M_ showed the highest degree of crystallinity at 70.67%, which demonstrates sharper diffraction with an intense peak at 22.6° compared to CNF-PP_1.0M_ and CNF-PP_3.8M_. Increases in the crystallinity of nanofibers are caused by hydronium ions’ (H_3_O^+^) charge on the reduction in the amorphous region during acid hydrolysis, leading to hydrolytic separation of the glycosidic bond of CNFs [21]. Compared to CNF-PP_1.0M_, CNF-PP_3.8M_ showed a slightly sharper peak at 22.6°. This is attributed to the fact that the separation and elimination of non-cellulosic components consisting of lignin, hemicellulose, and amorphous cellulose might occur in an amorphous region of the raw fibers, which leads to a rearrangement of the crystalline order in the crystallographic plane (200) [48]. These indicate that the acid hydrolysis pre-treatment had a significant impact on the crystalline regions of the cellulose nanofibers from Napier fibers, which was supported by the FTIR results. These results illustrate that the crystallinity of cellulose nanofibers can be altered accordingly, thus these cellulose nanofibers from Napier fibers have the potential to produce nanocomposite materials with a good reinforcement capabilities for various applications.

The crystallinity displayed in this study (CNF-PP_5.6M_ = 70.67%) was higher than the CNFs extracted by Mahardika et al. (69.4%) using high-shear homogenisation [46]. However, Syafri et al. showed a greater crystallinity than found in this study: 73.65% using chemical-ultrasonic treatment [49]. Nonetheless, when comparing to other studies, the present study provides a greater degree of crystallinity than those obtained by CNF from bagasse (68%) by Kord Sofla et al. [50], CNF from empty fruit bunches of oil palm (69%) by Jonoobi et al. [51] and CNF from banana peel (64.9%) by Pellissari et al. [23]. It can be concluded that a higher degree of crystallinity leads to higher tensile strength, thus enhancing the mechanical properties [48] of the nanofibers as durable reinforcement fillers.

### 3.4. FTIR Analysis

**Figure 4:**
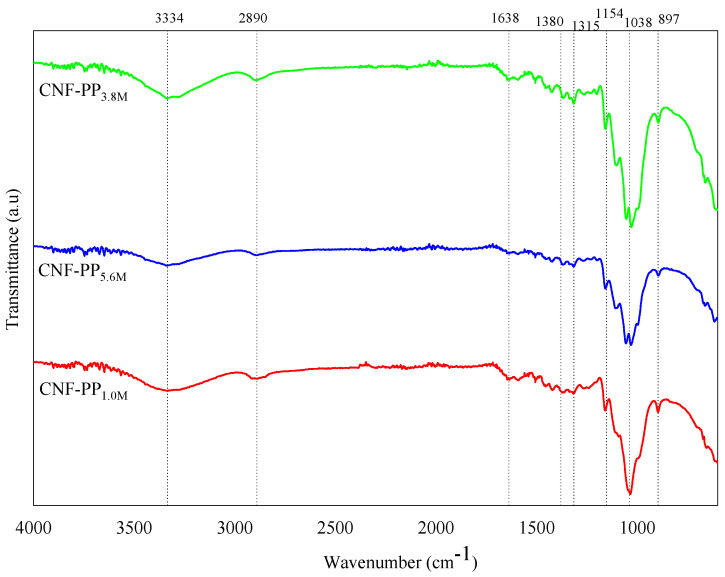
FTIR spectra of CNF-PP_1.0M_, CNF-PP_3.8M_ and CNF-PP_5.6M_.

The FTIR spectral features of CNF-PP_1.0M_, CNF-PP_3.8M_ and CNF-PP_5.6M_ under varying hydrolysis conditions are shown in Figure 4. Basically, the infrared spectral features revealed that there were no distinct changes observed among the functional groups of cellulose nanofibers of different molarity during acid hydrolysis. In addition, all the isolated nanofibers showed insignificant results on the I.R. spectra, which suggest that the isolation of CNFs at different molarities of acid hydrolysis did not influence the chemical composition of the samples. The finding concurs with the results of studies conducted on jute [7], Brazilian satin tail [52], and oil palm [53].

Figure 4 shows a broad band in the vicinity of 3334 cm^−1^ region for all samples, illustrates the presence of hydroxyl groups (O–H stretching vibration), which demonstrates the hydrophilic nature of the CNFs from Napier fibers. A more noticeable absorption peak in the spectra of the nanofibers was attained under CNF-PP_3.8M,_ whereas CNF-PP_5.6M_ is less intense owing to the exposure of cellulose in the high molarity of acid hydrolysis [52]. This is in good agreement with the observed agglomerations of CNF-PP_5.6M_ particles due to the presence of fewer hydroxyl groups, as mentioned in PSA analysis. Furthermore, the peak at 2890 cm^−1^ may be attributed to the C–H symmetrical stretching of cellulose and hemicellulose, and the band observed at 1638 cm^−1^ is the related to the O–H bending vibration of adsorbed water [54]. The absorbance band at 1380 cm^−1^ attributing to S = O stretching, indicating that sulphate group were presence during the acid hydrolysis where sulfuric acid was utilized as the catalyst. The peaks observed in the wavelength of 1315 cm^−1^ in all three samples could be associated with the bending vibrations and angular deformation of the C–H and C–O groups, corresponding to the polysaccharide aromatic ring [41]. The peak observed at 1154 cm^−1^ correspond to the C–O–C stretching vibration of the glucosidic rings [42] and the peak occurring at 1038 cm^−1^ is related to the C–O stretching of the pyranose ring vibration, referring to the structure of cellulose/lignin [52]. In addition, the increase in the crystallinity of the CNFs can be determined from the increase in intensity of this group [55]. Finally, the peaks at wavenumber 897 cm^−1^ in CNFs from Napier fibers are assigned to the β-glycosidic bonds that exist between the glucose units of cellulose/hemicellulose nanofibers. This band plays an important role in the CNF spectra, as it is proof that the cellulosic properties may not have been transformed during the acid hydrolysis process [56].

### 3.5. Thermogravimetric Analysis

**Figure 5:**
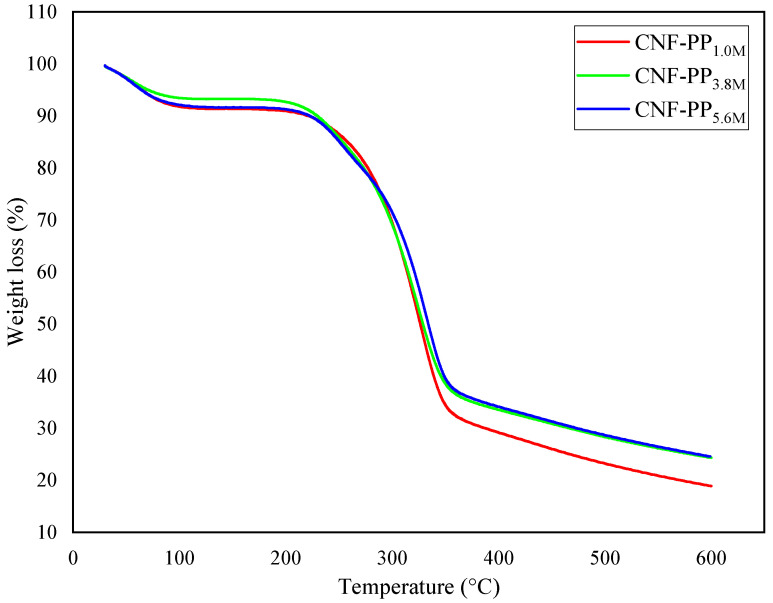
TGA curves of CNF-PP_1.0M_, CNF-PP_3.8M_ and CNF-PP_5.6M_.

**Figure 6:**
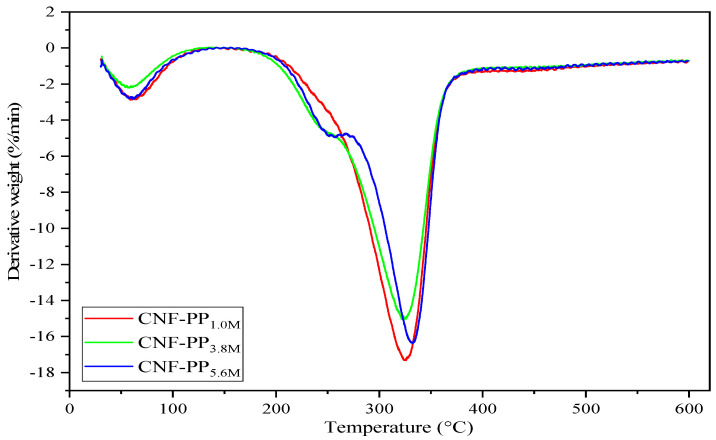
DTG curves of CNF-PP_1.0M_, CNF-PP_3.8M_ and CNF-PP_5.6M_.

Thermal decomposition and stability are essential factors to consider when proposing the use of CNFs as robust and natural fillers in reinforced composites. Figure 5 and Figure 6 represent the thermogravimetric analysis (TGA) and derivative thermogravimetric (DTG) curves of CNF-PP_1.0M_, CNF-PP_3.8M_ and CNF-PP_5.6M_, respectively. Two-step thermal decomposition behaviour was observed in this analysis; this is also known as a cellulose pyrolysis curve. This mechanism is strongly influenced by the chemical factors, presence of impurities, degree of crystallinity of cellulose, type of cellulose used, physical factors, temperature, heating time, and atmospheric conditions during the measurements [48]. The obtained thermal parameters of CNF-PP_1.0M_, CNF-PP_3.8M_ and CNF-PP_5.6M_ from TGA and DTG analyses are summarised in Table 4.

In the initial thermal degradation stage, the samples undergo weight loss due to dehydration [57]. These occurrences were observed between 30 and 100 °C, as displayed in Figure 5. On the other hand, samples with a lower molecular weight compound consisting of a hydroxyl group at the end of the chain might get evaporated at this stage, leading to further weight loss. Subsequently, the decomposition peak temperature presented in the DTG analysis illustrates a broad peak at 61.66, 58.07 and 62.50 °C for CNF-PP_1.0M_, CNF-PP_3.8M_ and CNF-PP_5.6M_, respectively, demonstrating that the evaporation of adsorbed water occurs in all CNF samples. The weight loss observed for this stage of degradation for all three samples CNF-PP_1.0M_, CNF-PP_3.8M_ and CNF-PP_5.6M_ was 8.17, 6.24 and 7.99%, respectively. Compared to CNF-PP_3.8M_ and CNF-PP_5.6M_, CNF-PP_1.0M_ displays a higher weight loss at temperatures ranging from 30 to 100 °C. Based on these results, it can be deduced that CNF-PP_1.0M_ possesses a higher moisture content than CNF-PP_3.8M_ and CNF-PP_5.6M_.

The second thermal degradation stage took place below 300 °C, between the approximate ranges of 140–280 °C, which acts as the most approachable region of the amorphous cellulose nanofibers, where there is a high content of sulfate groups. As seen in Table 4, CNF-PP_1.0M_, CNF-PP_3.8M_ and CNF-PP_5.6M_ started to decompose at temperatures of 269.74, 241.86 and 277.08 °C, respectively. According to Rahimi Kord Sofla et al., the thermal decomposition of cellulose begins in the amorphous regions and progresses to more crystalline regions [50]. These findings agree with the observations of Börjesson et al., [58] who observed that microcrystalline cellulose (MCC) with a prominent amorphous region is less resistant to high temperatures than those with ordered and compact crystalline structures. With regards to the decomposition temperature curve from the DTG analysis, as shown in Figure 6, the T_peak_ of CNF-PP_1.0M_, CNF-PP_3.8M_ and CNF-PP_5.6M_ were 324.90, 326.15 and 332.87 °C, respectively. This suggested that CNF-PP_5.6M_ has a better thermal stability than CNF-PP_1.0M_ and CNF-PP_3.8M_. In addition, CNF-PP_3.8M_ and CNF-PP_5.6M_ shows another significant step of thermal degradation at temperatures of approximately 250 °C. This step of thermal degradation can be referred to the existence of chemical additives which were utilized during the chemical treatments. Enhancement in the thermal stability of cellulose after undergoing chemical pre-treatment has been observed in past studies on samples such as chemically pre-treated wood fiber [59] and ramie [49].

The TGA curves illustrate that the increased molarities of acid hydrolysis lead to a decrease in the weight loss of the CNFs at temperatures above 300 °C, which is attributed to the improved thermal stability of the CNFs. However, the formation of char and low molecular weight gases occurred when active cellulose was dehydrated to produce anhydrocellulose, which consists of partially cross-linked cellulose particles [23]. The values of the char residue for CNF-PP_1.0M_, CNF-PP_3.8M_ and CNF-PP_5.6M_ at 600 °C were 18.91, 24.34 and 24.58%, respectively. CNF-PP_5.6M_ displayed similar residue content compared to CNF-PP_3.8M_ and a higher amount of residue content compared to CNF-PP_1.0M_, as shown in Table 4. This is likely due to the presence of sulphate groups in the hydrolysed CNFs, where sulphate groups act as flame retardants, increasing the content of charred residues at high temperatures [57].

## 4. Conclusions

In this study, cellulose nanofibers were isolated from Napier fibers via ball milling and acid hydrolysis at different molarities. The cellulosic properties of these CNFs may not have been transformed during the chemical and mechanical treatments, which is evident from the FTIR results. Furthermore, XRD analysis indicated that the CNFs isolated by acid hydrolysis with 5.6 M H_2_SO_4_ displayed the highest crystallinity value of 70.67%, indicating that it had the ability to perform well in the mechanical improvement of polymer nanocomposites. TGA revealed higher thermal stability for CNF-PP_5.6M_ than CNF-PP_1.0M_ and CNF-PP_3.8M_, which was probably caused by the reduction in residual cellulose in the amorphous region of the nanofiber, as well as an increase in crystallinity during the isolation process. The microscopy studies by AFM and TEM revealed that the isolation process leads to fibrillation and the breakage of fibers into nano-sized particles, which improves the effective surface area accessible for contact. FESEM results displayed a compact structure of the nanofiber network, due to the freeze-drying effect that resulted in the agglomeration of the nanofibers. Given these findings, it can be suggested that Napier fibers can be used to produce CNFs; to be employed as reinforcing materials for the development of nanocomposites in the biomedical, automotive, and pharmaceutical industries.

## Figures and Tables

**Table 1:** Selected processing parameters for the synthesis of cellulose nanofibers (CNFs) from Napier fibers.

Sample	Alkaline Pre-Treatment	Acid Hydrolysis Molarity	Alkaline Post-Treatment	Ball Milling		
				Time (Minutes)	Speed (rpm)	Ball Size (mm)
CNF-PP_1.0M_	12%	1.0 M	4%	180	840	15
CNF-PP_3.8M_	12%	3.8 M	4%	180	840	15
CNF-PP_5.6M_	12%	5.6 M	4%	180	840	15

**Table 2:** Diameters of CNF-PP_1.0M_, CNF-PP_3.8M,_ and CNF-PP_5.6M_ analysed by FESEM, TEM, and AFM microscopy.

Samples	FESEM (Diameter Size Range) (nm)	TEM (Diameter Size Range) (nm)	AFM (Diameter Size Range) (nm)
CNF-PP_1.0M_	49.93–167.60	5.04–90.67	26.44–192.50
CNF-PP_3.8M_	25.46–34.67	4.40–22.62	19.64–53.28
CNF-PP_5.6M_	16.10–34.95	5.67–13.70	10.50–38.74

**Table 3:** XRD analysis data of CNF-PP_1.0M_, CNF-PP_3.8M_ and CNF-PP_5.6M_.

Samples	Crystallinity Index, CrI (%)
CNF-PP_1.0M_	58.90
CNF-PP_3.8M_	65.18
CNF-PP_5.6M_	70.67

**Table 4:** Thermal parameters of CNF-PP_1.0M_, CNF-PP_3.8M_ and CNF-PP_5.6M_ obtained from thermogravimetric analysis (TGA) and derivative thermogravimetric (DTG) analysis.

Samples	1st Thermal Degradation			2nd Thermal Degradation			Residue at 600 °C (%)
	T_onset_ (°C) ^a^	T_peak_ (°C) ^b^	W_loss_ (%) ^c^	T_onset_ (°C) ^a^	T_peak_ (°C) ^b^	W_loss_ (%) ^c^	
CNF-PP_1.0M_	30.23	61.66	8.18	269.74	324.90	48.61	18.91
CNF-PP_3.8M_	30.40	58.07	6.24	241.86	326.15	50.46	24.34
CNF-PP_5.6M_	30.01	62.50	7.99	277.08	332.87	40.94	24.58

^a^ TGA onset temperature. ^b^ DTG decomposition peak temperature. ^c^ TGA weight loss.
